# Investigation of the methylation of Numb by the SET8 protein lysine methyltransferase

**DOI:** 10.1038/srep13813

**Published:** 2015-09-22

**Authors:** Sara Weirich, Denis Kusevic, Srikanth Kudithipudi, Albert Jeltsch

**Affiliations:** 1 Institute of Biochemistry, Stuttgart University, Pfaffenwaldring 55, 70569, Stuttgart, Germany

## Abstract

It has been reported that the Numb protein is methylated at lysine 158 and 163 and that this methylation is introduced by the SET8 protein lysine methyltransferase [Dhami *et al.*, (2013) Molecular Cell 50, 565–576]. We studied this methylation *in vitro* using peptide arrays and recombinant Numb protein as substrates. Numb peptides and protein were incubated with recombinant SET8 purified after expression in *E. coli* or human HEK293 cells. However, no methylation of Numb by SET8 was detectable. SET8 methylation of Histone H4 and p53 peptides and proteins, which were used as positive controls, was readily observed. While SET8 methylation of Numb in cells cannot be ruled out, based on our findings, more evidence is needed to support this claim. It appears likely that another not yet identified PKMT is responsible for the reported methylation of Numb in cells.

Histone post-translational modifications including acetylation, methylation and phosphorylation of several residues mainly in the histone tails are essential in modulating chromatin biology, gene expression and cellular development and they have important roles in diseases^1^,^2^,^3^. Histone lysine methylation can have various roles, for example H3K4 trimethylation is correlated with transcriptional activation, while H3K9 and H3K27 trimethylation correlates with transcriptional repression. H4K20 is subjected to mono, di- and trimethylation and different methylation states are introduced by various enzymes^4^,^5^. The SET8/Pr-Set7/KMT5a protein lysine methyltransferase (PKMT) introduces H4K20 monomethylation^6^,^7^, which further acts as substrate for SUV4-20H1 and SUV4-20H2 for introducing di- and trimethylation^8^. H4K20 monomethylation plays an important role in cell cycle control and genome stability, and H4K20 trimethylation is associated with heterochromatin formation and gene repression^4^,^5^,^9^. The active center of SET8 is located in its SET (Su(var)3–9, Enhancer-of-zeste and Trithorax) domain^10^. Different studies documented that SET8 acts as monomethyltransferase introducing one methyl group on an unmethylated substrate lysine residue^11^,^12^,^13^. Similar to SET7/9, another well-characterized monomethyltransferase^14^,^15^, the active pocket of SET8 is surrounded by tyrosine residues including Y334, which forms hydrogen bonds with the ε−amino group of lysine and prevents higher degrees of methylation. When this residue was exchanged to phenylalanine the mutated SET8 could introduce dimethylation at H4K20^11^. We along with others have shown that SET8 is a highly specific PKMT, which recognizes a long peptide sequence between R17 and R23 on H4 [R^17^-H^18^-(R^19^KY)-K^20^-(V^21^ILFY)-(L^22^FY)-R^23^]^11^,^13^. Like other PKMTs, SET8 was found to methylate non-histone proteins and peptide substrates as well^13^,^16^. The first identified non-histone protein substrate of SET8 was the tumor suppressor protein p53. SET8 mediated mono-methylation of p53 at K382 promotes the methyl specific interaction of p53 with the MBT domains of L3MBTL1 protein which mediates the repression of p53 target genes^16^,^17^. Recently, it was reported that SET8 dimethylates the Numb protein at K158 and K163^18^. Numb methylation at these residues was shown to disrupt its interaction with p53 and the unbound p53 eventually became ubiquitinated, which led to a reduction of apoptosis^18^. However, since SET8 is a very specific PKMT with a long recognition sequence and it was found previously to function as a monomethyltransferase, we were interested to confirm that purified SET8 can dimethylate Numb peptides and protein *in vitro*.

## Results

**Figure 1:**
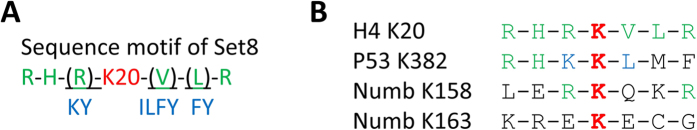
Peptide substrate sequence motif of SET8 and sequence alignment of histone H4, p53 and Numb target lysines. (**A**) The substrate specificity profile of SET8 was adopted from Kudithipudi *et al.* (2012)^13^. The upper row represents the amino acid sequence of the H4 tail surrounding K20 (printed in green), the lower row specifies the other amino acids that are also accepted at the corresponding position (printed in blue). (**B**) Sequence alignment of the H4, p53 and Numb target lysine residues. The target lysine is in red. Green and blue coloring is as indicated in A.

Previously, our lab has identified the substrate peptide motif of SET8^13^ by analyzing specificity profile peptide arrays using the H4 sequence as template. Our data showed that SET8 has a long recognition motif, because it reads R17 to R23 of the H4 tail and accepts only few alternative residues mainly at the −1, +1 and +2 positions (considering the target lysine as position 0) (Fig. 1A). Peptide array experiments have shown that exchanges of single amino acids within this region can abrogate catalytic activity if a disfavored residue is introduced^13^. However, the sequence alignment of the lysine residues methylated in Numb (K158 and K163) with the SET8 reported substrates p53-K382 and H4K20 shows only limited sequence similarity and the reported Numb methylation sites differ at least at 4 out of 6 position from the SET8 preference (Fig. 1B). Therefore, we were interested to validate Numb methylation by SET8.

### Methylation analysis of Numb peptides

**Figure 2:**
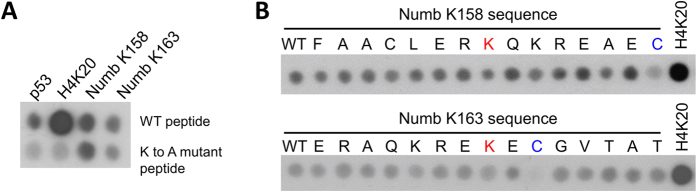
Peptide array methylation. (**A**) Methylation activity of SET8 on peptides containing the previously reported target lysines of p53, H4 and Numb. The upper row contains the wild type peptides (WT) and the lower row the same peptides with the target lysine exchanged to alanine. List of the peptide sequences: p53 (SRHKKLMFKTEGPDS), H4K20 (GGAKRHRKVLRDNIQ), Numb K158 (FAACLERKQKREKEC), Numb K163 (ERKQKREKECGVTAT) (the target lysine is underlined). (**B**) Sequence scan arrays of the Numb K158 and Numb K163 peptides methylated by SET8 to identify the sites of residual methylation. All 15 amino acids of both peptides were individually exchanged to alanine or serine (in case of an alanine at the place). In addition, the second putative target lysine was also exchanged by alanine (K158 in the K163 peptide and vice versa). The H4K20 peptide was included as positive control. In both cases, the exchange of the target lysine(s) (labelled in red) did not reduce methylation strongly, but methylation was almost lost after exchange of C165 (labelled in blue).

To investigate if SET8 can methylate Numb peptides *in vitro*, we used peptide arrays synthesized on cellulose membrane as substrates. SET8 was overexpressed in *E. coli* BL21 cells and purified by affinity chromatography. The peptide arrays were synthesized with 15 amino acid long peptides containing the target lysine at the center. Numb peptides with the sequences surrounding K158 and K163 were used and in addition control peptides in which the target lysines were exchanged by alanine. H4K20 and p53K382 peptides were included as positive controls and corresponding target lysine variants as negative controls. The peptide arrays were incubated with SET8 in the presence of radioactively labeled AdoMet and the transfer of methyl groups to the immobilized peptides was detected by autoradiography (Fig. 2A). As expected, methylation signals were observed on the p53 and H4K20 wild type peptides, which were lost on the corresponding peptides with target lysine exchange indicating a specific methylation of the target lysine. With the Numb K158 and Numb K163 peptides, a weak radioactive signal was observed which was comparable in intensity to the methylation of the p53 peptide. However the methylation signals of the Numb peptides did not change, even when the target lysines were mutated to alanine (Fig. 2A). This result indicates that the signal at the Numb peptides either originates from binding of SET8-AdoMet complexes to these peptides or that another amino acid residue is methylated. In total, we observed absence of SET8 introduced lysine methylation of Numb peptides in 7 independent peptide array methylation experiments in which positive control peptides were methylated as expected.

To further investigate the signals appearing on the Numb peptides, we synthesized mutational scanning peptide arrays of the Numb (151–165) and Numb (156–170) peptides in which each individual residue was altered to alanine (or serine if the original residue was alanine) (Fig. 2B). The H4K20 peptide was included as positive control. The peptide arrays were methylated with SET8 in the presence of radioactively labeled AdoMet as described above. In agreement with the previous results, the strongest methylation signal was observed with the H4K20 peptide and weak methylation signals were observed with most of the Numb peptides. Exchange of any of the lysine or arginine residues in these peptides did not cause a strong reduction in signal, as it would have been expected, if these residues were methylated. Instead, we observed a strong loss of signal with peptides which carry a C165A mutation. This result suggests that this cysteine is the target amino acid of SET8 for methylation in the Numb peptides (Fig. 2B). Weak cysteine methylation by SET8 is not surprising since cysteine is the strongest nucleophile among all amino acid residues and cysteine methylation has been reported before for other AdoMet dependent methyltransferases, for example MLL1^19^ or DNMT3A^20^. In summary these results show that recombinant SET8 expressed in *E. coli* cannot methylate the Numb peptides on peptide arrays while positive control peptides were methylated as expected.

### Methylation analysis of the Numb protein with SET8 purified from E. coli

**Figure 3:**
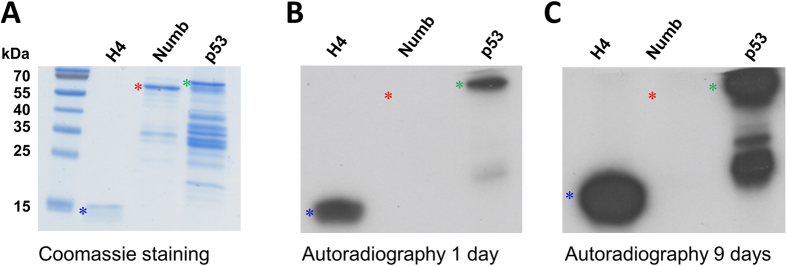
*In vitro* protein methylation of Numb and other proteins used as positive controls by recombinant SET8 purified from *E. coli*. (**A**) Coomassie BB stained SDS polyacrylamide gel of the purified GST-Numb, GST-p53 and recombinant H4 used as methylation substrates. The corresponding bands of H4, GST-Numb and GST-p53 are labelled by blue, red and green asterisks. (**B**) Methylation of GST-Numb, GST-p53 and H4 with recombinant SET8. The positions of the corresponding protein bands are indicated as described in (**A**). Methylation signals from H4 and GST-p53 were detected, whereas no methylation signal was visible for GST-Numb. (**C**) Same image as in (**B**) but after longer exposition as indicated.

Next, we investigated the ability of SET8 to methylate the Numb protein. For this we cloned the Numb protein domain (residues 12–272) as GST fusion. The GST-Numb protein domain was overexpressed in *E. coli* and purified by affinity chromatography. Methylation reactions were performed by incubating the substrate protein with SET8 in methylation buffer containing radioactively labeled AdoMet. Histone H4 and GST-p53 domain (residues 20–372) were included as positive controls. To ensure that comparable amounts of proteins were used for methylation, all purified proteins were analyzed by 16% SDS-PAGE (Fig. 3A). The methylated samples were loaded on an SDS PAGE and the transfer of methyl groups was detected by autoradiography. As expected, we observed clear methylation signals with the positive controls H4 and p53. However, no methylation of Numb was detected (Fig. 3B). This result indicates that recombinant SET8 expressed in *E. coli* cannot methylate the Numb protein *in vitro*. A semi-quantitative analysis of a longer exposition of the same image (Fig. 3C) indicates that a putative residual methylation activity of SET8 on Numb must be smaller than 0.1% or 0.01% of the activity observed with p53 or H4, respectively.

### Methylation analysis of the Numb protein with recombinant SET8 purified from human cells

**Figure 4:**
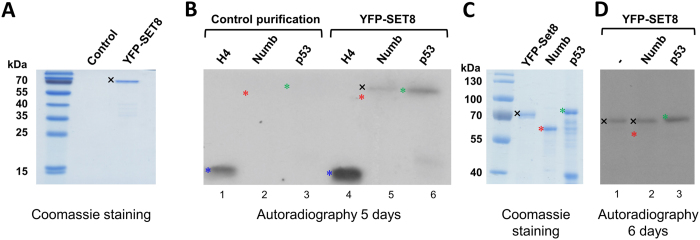
*In vitro* protein methylation of Numb and p53 by YFP-SET8 purified from human cells and automethylation of SET8. (**A**) Coomassie BB stained SDS polyacrylamide gel of YFP-SET8 purified from HEK293 cells after ectopic expression (labelled with a black cross). As a control, the same purification was carried out from HEK293 cells without YFP-SET8 expression. (**B**) Methylation of GST-Numb, GST-p53 and H4 with YFP-SET8 purified from HEK293 cells (lanes 4–6). The corresponding bands of H4, GST-Numb, GST-p53 and YFP-SET8 are labelled by blue, red and green asterisks and a black cross. Methylation of H4 and GST-p53 was detected, no methylation was observed for GST-Numb. The weak band that appears slightly higher than p53 in the GST-Numb sample (labelled with a black cross) corresponds to automethylation of SET8. In the reactions with the control purification (lanes 1–3) only a weaker methylation of H4 was observed. The corresponding loading control for the substrate proteins is shown in Fig. 3A. (**C**) Coomassie BB stained SDS polyacrylamide gel of purified YFP-SET8 from HEK293 cells after ectopic expression, and purified GST-Numb and GST-p53 proteins used as methylation substrates. The corresponding bands of GST-Numb and GST-p53 are labelled as in panel (**A**). The image shown here represents the loading of the proteins used in the methylation assays shown in panel (**D**). (**D**) Incubation YFP-SET8 purified from HEK293 cells without external substrate and with GST-Numb or GST-p53 (lanes 1–3) in the presence of radioactively labeled AdoMet. The autoradiography image shows automethylation of SET8 (marked with the black cross) in the lanes 1 and 2. No methylation was detected for the Numb protein (marked with red star in lane 2). The p53 protein shows a clear methylation signal (marked with green star in lane 3).

Dhami *et al.* (2013) performed *in vitro* methylation reactions of the Numb protein with recombinant SET8 protein purified from human cells by immune precipitation^18^. Since post-translational modifications or interactors of SET8 still present in the preparation may modulate the enzyme’s activity or specificity, we next aimed to investigate the ability of recombinant SET8 purified from HEK293 cells to methylate the Numb protein. To this end, ectopically expressed YFP-SET8 was immunoprecipitated from HEK293 cell extract using GFP-Trap. An identical mock purification was performed with untransfected HEK293 cells and used as a negative control. The resulting preparations were incubated individually with histone H4, Numb and p53 in the presence of radioactively labeled AdoMet. The methylation mixture was separated on an SDS polyacrylamide gel and the methylation signal was detected by autoradiography (Fig. 4B). With YFP-SET8 a strong methylation signal was observed with H4 and weaker methylation of p53 was detected as well. In the control reactions, the H4 methylation signal was much weaker and there was no detectable p53 methylation signal, indicating that some endogenous PKMT activity was co-purified. However, comparison of the intensities indicates that the activity detected after incubation of the target proteins with the purified YFP-SET8 was mostly (H4) or exclusively (p53) due to the purified SET8. After incubation of Numb with the purified YFP-SET8, a weak signal was observed, but this band did not run at the size expected for Numb (slightly below p53) but at the size of YFP-SET8, suggesting that it corresponds to automethylation of SET8, which is a common side-reaction of PKMTs^21^,^22^,^23^ and PRMTs^24^,^25^,^26^.

To confirm the automethylation of SET8, we repeated the protein methylation experiments with the cellular purified YFP-SET8. Like before, GST-Numb and GST-p53 proteins were incubated with YFP-SET8 in presence of radioactively labeled AdoMet, but, in addition, YFP-SET8 protein was incubated under the same reaction conditions without external protein substrate. Afterwards, the protein methylation was detected by autoradiography (Fig. 4D). In agreement with the previous results, methylation of GST-p53 was readily detected (marked with a green star). In addition, a weak signal appeared in the YFP-SET8 and Numb protein lanes at the size corresponding to the YFP-SET8 protein (marked in black cross) which confirms that automethylation is happening. As shown previously, no methylation signal was observed at the position of the Numb protein (marked by a red star). In summary, we conclude that neither the recombinant SET8 protein purified from the *E. coli* nor recombinant SET8 purified from mammalian cells is able to methylate the Numb protein *in vitro*.

## Discussion

Recently, Dhami *et al.* (2013) identified in cellular studies dimethylation of the Numb protein at K158 and K163 and they showed that this has important biological consequences^18^. Given the essential biological role of Numb methylation it is imperative to identify the PKMT enzyme responsible for this modification. Dhami *et al.* (2013) provided evidence that SET8 introduces this methylation. We realized that the Numb methylation sites differ strongly from the SET8 substrate preferences^11^,^13^ and conducted experimental studies to validate Numb methylation by SET8. However, our results show that SET8 is not able to methylate the Numb protein at these two lysine residues *in vitro*.

The original claim of Dhami *et al.* (2013) that SET8 methylates Numb was mainly based on the observation that co-expression of SET8 and Numb in cells increased Numb methylation. In addition, overexpression of SET8 reduced p53 binding of Numb (because methylated Numb does not interact with p53), while SET8 knock-down augmented Numb p53 binding. One caveat of such cellular studies is that it is difficult to distinguish direct and indirect effects, for example overexpression or knock-down of SET8 may have caused a change in the activity of another PKMT, which introduced methylation of Numb. In addition, Dhami *et al.* (2013) conducted *in vitro* methylation experiments of Numb peptides with SET8 immunopurified from cells. However, these data were inconclusive, because the purity of the immuoprecipitated SET8 was not shown and the absence of contaminating PKMTs was not demonstrated, e.g. by using a catalytically inactive SET8 as a negative control or doing a mock purification. Methylation was analyzed by mass spectrometry, but only MS/MS data were shown, which do not allow to assess the level of methylation. Moreover, the spectra were not fully convincing, because the intensity coverage of the fit of the spectra to the theoretical Numb peptide fragments was poor. No methylation data with H4 or p53 were shown as positive reference to estimate the relative efficiency of Numb methylation by SET8.

Lack of *in vitro* activity as observed here by us could be caused by a failure to purify active enzymes or wrong reaction conditions. However, in our study the use of positive controls rules out these trivial explanations for the lack of Numb methylation by SET8. Evidently, lack of *in vitro* activity cannot rule out activity in cells. For example, it is conceivable, that cellular interaction partners, PTMs or other cues may alter the substrate spectrum of the PKMT and by this hypothetically may allow SET8 to methylate Numb in cells. This assumption is plausible for enzymes with little readout of the target peptide sequence (like SET7/9^15^ or PRMTs^27^), which contain a rather “universal” catalytic pocket and select the cellular methylation substrates by protein/protein interactions at other interfaces. However, SET8 forms many specific contacts to residues of the substrate peptide or protein which mediate a very specific sequence readout. So far, there is no precedence case in the PKMT field, where a highly specific enzyme has massively changed its specificity by an interaction with another protein or after introduction of a PTM. Moreover, we show here that SET8 purified from human cells is not able to methylate Numb. Finally, the dimethylation of Numb also sheds doubts on the claim that SET8 is the responsible PKMT, because SET8 so far was described as monomethyltransferase^11^,^12^,^13^. We conclude that SET8 methylation of Numb in cells cannot be ruled out, but more evidence is needed to support this claim. Currently, it appears more likely that another not yet identified PKMT is responsible for the detected Numb methylation in cells.

## Methods

### Cloning expression and purification of proteins

The coding sequence of the Numb protein (residues 12–272; UniprotKB entry P49757) was amplified from cDNA derived from HEK293 cells and cloned as GST fusion protein into pGEX-6P-2 (GE Healthcare). For protein expression, *E. coli* BL21 cells (Novagen) were transformed with the corresponding plasmids and grown in Luria-Bertani media at 37 °C, until they reached an optical density of 0.6 to 0.8 at 600 nm. The cells were transferred to 20 °C and then induced with 1 mM isopropoyl-beta-D-thiogalactopyranoside and grown for 10–12 h. The GST fusion proteins were purified as described before^15^. The SET domain of the SET8 protein (residues 114–352; UniprotKB entry Q9NQR1) and p53 (residues 20–372; UniprotKB entry P04637) were expressed and purified as described before^13^. Recombinant H4 was purchased from New England Biolabs.

### Synthesis of peptide SPOT arrays

Peptide arrays were synthesized on cellulose membrane by the SPOT synthesis method^28^ using a Multipep system (Intavis AG). Each spot contained approximately 9 nmol peptide (Autospot Reference Handbook, Intavis AG). Successful synthesis of peptides on the cellulose membranes was confirmed by bromophenol blue staining.

### Methylation of peptide arrays

The peptide arrays were pre-incubated for 10 min in methylation buffer (20 mM HEPES pH8, 50 mM NaCl, 5 mM DTT) and then incubated in the methylation buffer containing 300 nM SET8 and 0.76 μM labeled [methyl-^3^H]-AdoMet for 60 min. The membranes were washed 5 times for 5 minutes with wash buffer (100 mM NH_4_HCO_3_ and 1% SDS). Finally, the peptide arrays were incubated with Amplify NAMP100V solution (GE Healthcare, Munich, Germany) for 5 minutes. The peptide arrays were exposed to Hyperfilm TM high performance autoradiography film (GE Healthcare, Munich, Germany) in the dark at −80 °C for 7–10 days. The autoradiography films were developed in Optimus TR developing machine.

### Protein methylation reactions

Protein methylation was performed by incubating the substrate proteins in methylation buffer containing 20 mM HEPES pH 8, 50 mM NaCl, 5 mM DTT supplemented with 0,76 μM labeled [methyl-^3^H] AdoMet and 500 nM of SET8 for 3 h at room temperature. Afterwards the methylation reaction was stopped by boiling with SDS loading buffer and the reaction mixture loaded on a 16% SDS PAGE. The methylation signal was detected by autoradiography.

### Cell culture, transfection and immunoprecipitation

HEK293 cells were grown in Dulbecco´s Modified Eagle´s Medium (Sigma) supplemented with 5% fetal bovine serum, penicillin/streptomycin and L-glutamine (Sigma). The YFP-SET8 plasmid was transfected into cells using polyethylenamine (Promega, according to the manufacturer´s instructions). 48 h after transfection cells were washed with PBS buffer and harvested by centrifuging at 525 g for 5 min. The YFP-SET8 fusion protein was immunoprecipitated from mammalian cell extract using GFP-Trap A (Chromotek) following the manufacturer’s instructions.

## Additional Information

**How to cite this article**: Weirich, S. *et al.* Investigation of the methylation of Numb by the SET8 protein lysine methyltransferase. *Sci. Rep.* **5**, 13813; doi: 10.1038/srep13813 (2015).
